# Perceived infection transmission routes, infection control practices, psychosocial changes, and management of COVID-19 infected healthcare workers in a tertiary acute care hospital in Wuhan: a cross-sectional survey

**DOI:** 10.1186/s40779-020-00254-8

**Published:** 2020-05-11

**Authors:** Ying-Hui Jin, Qiao Huang, Yun-Yun Wang, Xian-Tao Zeng, Li-Sha Luo, Zhen-Yu Pan, Yu-Feng Yuan, Zhi-Min Chen, Zhen-Shun Cheng, Xing Huang, Na Wang, Bing-Hui Li, Hao Zi, Ming-Juan Zhao, Lin-Lu Ma, Tong Deng, Ying Wang, Xing-Huan Wang

**Affiliations:** 1 Center for Evidence-Based and Translational Medicine, Zhongnan Hospital of Wuhan University, 169 Donghu Road, Wuchang District, Wuhan, 430071 Hubei China; 2 Department of Evidence-Based Medicine and Clinical Epidemiology, The Second Clinical College, Wuhan University, Wuhan, 430071 Hubei China; 3 Division of Medical Affairs, Zhongnan Hospital of Wuhan University, Wuhan, 430071 Hubei China; 4 Department of Hepatopancreatobiliary Surgery, Zhongnan Hospital of Wuhan University, Wuhan, 430071 Hubei China; 5 Division of Social and Medical Development, Zhongnan Hospital of Wuhan University, Wuhan, 430071 Hubei China; 6 Department of Respiratory Medicine, Zhongnan Hospital of Wuhan University, Wuhan, 430071 Hubei China; 7 School of Nursing and Health, Henan University, Kaifeng, 475000 Henan China; 8 Institute of Evidence-Based Medicine and Knowledge Translation, Henan University, Kaifeng, 475000 Henan China; 9 Department of Infectious Diseases, Zhongnan Hospital of Wuhan University, Wuhan, 430071 Hubei China; 10 Leishenshan Hospital in Wuhan, Wuhan, 430200 Hubei China

**Keywords:** COVID-19, SARS-CoV-2, 2019-nCoV, Healthcare worker, Healthcare professional, Infection transmission route, Psychosocial status

## Abstract

**Background:**

Many healthcare workers were infected by coronavirus disease 2019 (COVID-19) early in the epidemic posing a big challenge for epidemic control. Hence, this study aims to explore perceived infection routes, influencing factors, psychosocial changes, and management procedures for COVID-19 infected healthcare workers.

**Methods:**

This is a cross-sectional, single hospital-based study. We recruited all 105 confirmed COVID-19 healthcare workers in the Zhongnan Hospital of Wuhan University from February 15 to 29, 2020. All participants completed a validated questionnaire. Electronic consent was obtained from all participants. Perceived causes of infection, infection prevention, control knowledge and behaviour, psychological changes, symptoms and treatment were measured.

**Results:**

Finally, 103 professional staff with COVID-19 finished the questionnaire and was included (response rate: 98.1%). Of them, 87 cases (84.5%) thought they were infected in working environment in hospital, one (1.0%) thought their infection was due to the laboratory environment, and 5 (4.9%) thought they were infected in daily life or community environment. Swab of throat collection and physical examination were the procedures perceived as most likely causing their infection by nurses and doctors respectively. Forty-three (41.8%) thought their infection was related to protective equipment, utilization of common equipment (masks and gloves). The top three first symptoms displayed before diagnosis were fever (41.8%), lethargy (33.0%) and muscle aches (30.1%). After diagnosis, 88.3% staff experienced psychological stress or emotional changes during their isolation period, only 11.7% had almost no emotional changes. Arbidol (Umifenovir; an anti-influza drug; 69.2%) was the drug most commonly used to target infection in mild and moderate symptoms.

**Conclusion:**

The main perceived mode of transmission was not maintaining protection when working at a close distance and having intimate contact with infected cases. Positive psychological intervention is necessary.

## Background

Infection in medical institutions happens easily when a new epidemic occurs. According to WHO daily situation report, after the coronavirus disease 2019 (COVID-19) outbreak, 22,073 COVID-19 cases among healthcare workers have been reported to the WHO as of Wednesday, 8 April 2020 [1]. The number of healthcare workers infected by severe acute respiratory syndrome coronavirus 2 (SARS-CoV-2) reached 1716 China wide on February 11, 2020; among them, 1502 cases were in Hubei province; with 1102 in Wuhan [2]. As of early March, the infected number increased to 3300 and at least 22 died in China, and there were over 2600 infected with 13 died in Italy as of 20 March, 2020 [3–5]. Reports from medical staff describe physical and mental exhaustion, the torment of difficult triage decisions, and the pain of losing their patients and colleagues, all in addition to the infectious risk. Healthcare workers are the pillar for fighting against this pandemic; hence, preventing them to be infected is a big challenge for maintaining a strong fighting force, high morale, and energy for fighting. Now to protect healthcare workers away from infection has aroused great concern, many experts published opinions [3, 4, 6–10] to appeal to worldwide for paying attention to protect health care workers from woefully unprepared status.

On 2 March 2020, Lai et al. [11] reported mental health of 1257 healthcare workers treating patients exposed to COVID-19 in 34 hospitals in China. Results showed that they experienced psychological burden, especially nurses, women, those in Wuhan, and frontline healthcare workers directly engaged in the diagnosis, treatment, and care for patients with COVID-19. Another qualitative study released 18 February 2020 suggested that maintains healthcare workers’ mental health is essential to better control infectious diseases [10]. Obviously, there is no study concerned about the infected healthcare workers with COVID-19 and their mental health. Therefore, we conducted this epidemiological investigation and aimed to explore the route of SARS-CoV-2 infection, influence, and management procedures of healthcare workers. We also believe our work will provide factual evidence and lessons learned can be used to prevent such infections in the future.

## Methods

### Study design

This is a cross-sectional and single center study, using data of all 105 infectious healthcare staff from Zhongnan Hospital of Wuhan University. 105 healthcare staff became infected before January 30, 2020, our study period was from February 15 to 29, 2020; and no new staff became infected until now. This study was reviewed and approved by the Committee for Ethical Affairs of this hospital.

All 105 healthcare professionals who were recorded in the Division of Medical Affairs of confirmed COVID-19 diagnosis were invited to finish the questionnaire. Confirmation diagnosis was according to the diagnostic criteria of the National Health Committee of the People’s Republic of China (CNHC) [12].

**Fig. 1:**
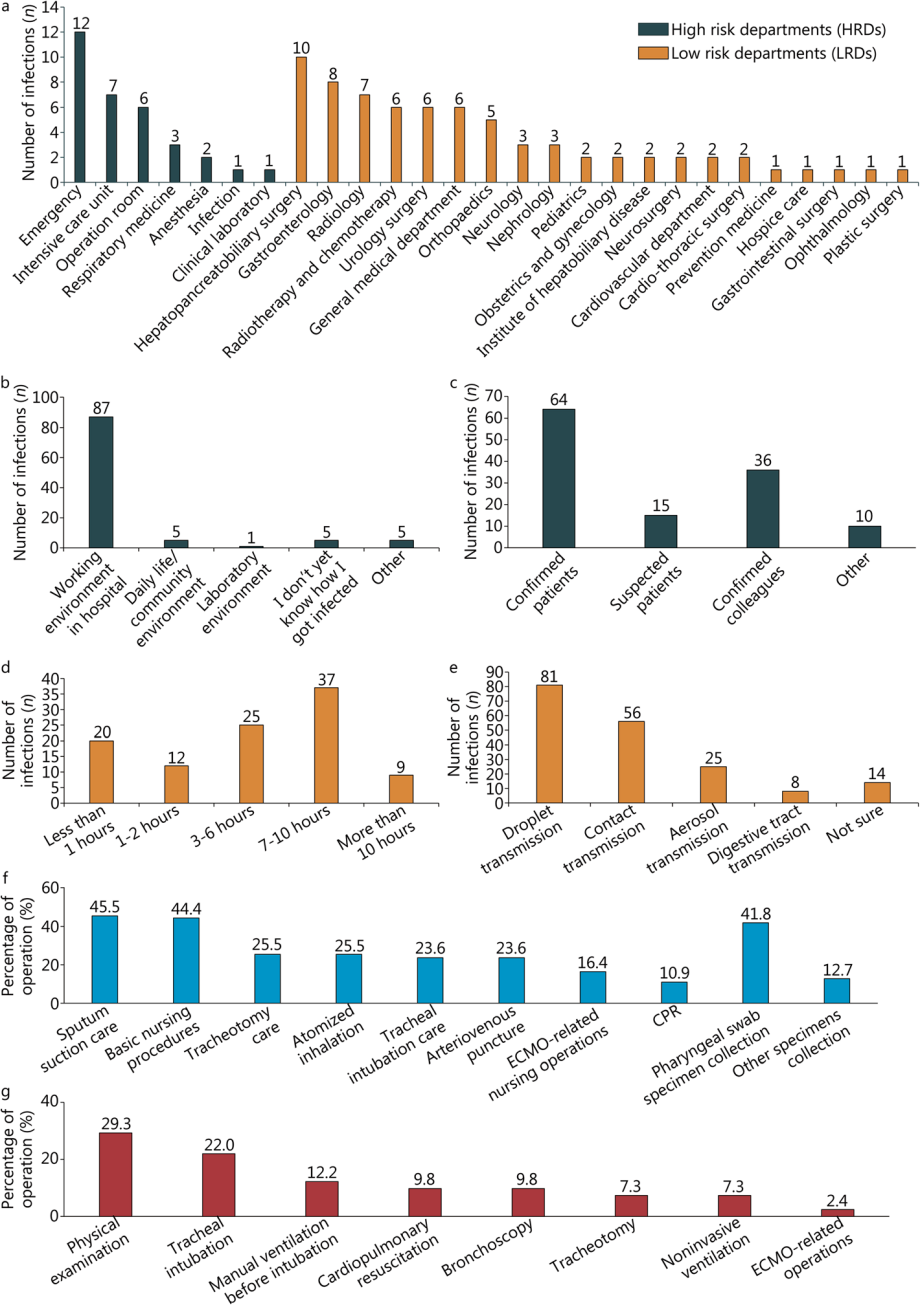
Perceived causes of COVID-19 infection in medical professionals **a**. Department distribution of infected healthcare professionals; **b**. Perceived routes of infection; **c**. Perceived routes of infection in working environment in hospital; **d**. Duration distribution in an environment with risk of infection; **e**. Perceived transmission routes; **f**. Perceived infection causing procedures among nurses; **g**. Perceived infection causing procedures among doctors

We divided the departments represented in this study into two groups: high risk of nosocomial infection departments (HRDs) and low risk of nosocomial infection departments (LRDs) according to general principles for the management of nosocomial infection control of CNHC [13] and infection control expert opinion from this project group. HRDs included departments of respiratory medicine, infectious diseases, emergency, clinical laboratory, anesthesia surgery, operating room, and intensive care unit; all other departments were categorized as LRDs (Fig. 1a). All staffs were working in their normal departments and no one had been redeployed to other areas at the time of the survey.

### Measuring instruments and data collection

We employed a self-administered electronic tool to collect data using our validated questionnaire which included informed consent (see Additional file 1). After readability and content validity test, the test-retest reliability coefficient of our electronically administered questionnaire was 0.82 (1 = perfect repeatability) [14]. We also cross-checked information was from other sources. Epidemiologic data were confirmed through phone calls to all participants, and we checked electronic patient records for collecting treatment information. Additionally, our investigators also held tele-interviews with the department directors of infected ones where necessary, to determine exposure status of all staff in the week before SARS-CoV-2 infection.

### Framework

We conceptualized a research framework initially based on the following: 1) Online interview or a free response set of questions to explore infection prevention and control experiences of COVID-19 among frontline clinicians from the office of nosocomial infection control. 2) Policy documents, guidelines or consensus for this epidemic or general prevention of infectious disease, such as notice on the issuance of a program for prevention and control of SARS-CoV-2 infection from the CNHC.

### Survey sampling, questionnaire administration

Samples presented were all from Zhongnan Hospital of Wuhan University. Zhongnan Hospital of Wuhan University is a 3500 bedded tertiary acute teaching hospital which has more than 800 doctors and 1800 nurses. This hospital is equipped with 46 clinical and medical technology departments, 17 teaching and research departments and 1 laboratory. Zhongnan Hospital of Wuhan University is one of the key hospitals at the epicentre of this outbreak, and had treated 952 hospitalized patients with COVID-19 between January 1 and March 12. Samples selection in this study was not limited by departments of professional staff. We contacted all infected professional staff according to the hospital’s records (*n =* 105). Electronic questionnaires were sent to all infected staff member and send a message by mobile phone to reminder, and they were free to choose whether take part in or not; if unwilling, the can click “do not agree to participate in this research” bottom (see Additional file 1).

### Statistical analysis

Categorical variables were described as frequencies and percentages, continuous variables were showed as Mean ± standard deviation (SD) or median (25–75% percentile) as appropriate. To assess the changes before and after COVID-19 outbreak, McNemar tests were performed for the change of training level, Wilcoxon signed rank sum tests were conducted for the mastery level for knowledge of protective procedures which were measured using a Likert scale. All analyses were carried out using the SAS software (version 9.4 TS1M6, SAS Institute Inc., Cary, NC, US). Two-sided *P* values less than 0.05 were considered statistically significant. The data were visualized by Microsoft 2019.

## Results

### Basic information

All 105 infected medical staff with COVID-19 in our hospital was invited, finally 103 cases agreed with the consent and finished our questionnaire was included for analysis (response rate: 98.1%), Table 1 presented their basic characteristics. Of them, 71 worked in LRDs and 32 in HRDs (Fig. 1a), the median age was 35.0 years, 41 were doctors (39.8%), 55 were nurses (53.4%), and 7 were medical technicians (6.8%). 39 (37.9%) were males and 64 (62.1%) were females.

**Table 1:** Baseline characteristics of medical staff infected with COVID-19

Characteristic	Total (*n =* 103)	LRDs (*n =* 71)	HRDs (*n =* 32)
Male [*n* (%)]	39(37.9)	28(39.4)	11(34.4)
Age [years, median (IQR)]	35.00(14.0)	36.00(17.0)	32.00(12.0)
22 ~ 30	32(31.1)	19(26.8)	13(40.6)
31 ~ 40	40(38.8)	26(36.6)	14(43.8)
41 ~ 50	16(15.5)	11(15.5)	5(15.6)
51 ~ 62	15(14.6)	15(21.13)	0(0)
BMI [kg/m^2^, mean ± SD]	22.18 ± 2.81	22.12 ± 3.01	22.32 ± 2.35
< 18.5	9(8.7)	8(10.8)	2(4.8)
18.5 ~ 24	67(65.1)	44(59.5)	31(73.8)
≥ 24	27(26.2)	22(29.7)	9(21.4)
Marital status [*n* (%)]			
Married	77(74.8)	55(77.5)	22(68.8)
Single	25(24.3)	15(21.1)	10(31.3)
Divorced	1(1.0)	1(1.4)	0(0.00)
Occupations [*n* (%)]			
Doctor	41(39.8)	28(39.4)	13(40.6)
Nurse	55(53.4)	37(52.1)	18(56.2)
Medical technician	7(6.8)	6(8.5)	1(3.1)
Work experience [median (IQR), years]	9.00(19.0)	10.00(23.0)	7.50(13.0)
Work capacity/level [*n* (%)]			
Senior level	12(11.7)	11(15.5)	1(3.1)
Associate Senior level	16(15.5)	13(18.3)	3(9.4)
Intermediate level	29(28.2)	17(23.9)	12(37.5)
Junior level	46(44.7)	30(42.3)	16(50.0)
Smoking [*n* (%)]			
Yes	5(4.9)	4(5.6)	1(3.1)
No	94(91.3)	64(90.1)	30(93.8)
Quit smoking	4(3.9)	3(4.2)	1(3.1)
Drinking [*n* (%)]			
Yes	9(8.7)	8(11.3)	1(3.1)
No	90(87.4)	60(84.5)	30(93.8)
Quit drinking	4(3.9)	3(4.2)	1(3.1)
Experience in treatment and nursing (e.g. SARS) [*n* (%)]	5(4.9)	1(1.4)	4(12.5)

*IQR* Inter quartile range, *BMI* Body mass index, *LRDs* Low risk of nosocomial infection department, *HRDs* High risk of nosocomial infection departments

### Causes of infection

#### Route of infection

Figure 1b presented the perceived routes of infection. Among the 103 responders, 87 (84.5%) cases thought they were infected by the working environment in hospital, one (1.0%) thought infection was due to the laboratory environment with biological specimens of suspected or confirmed patients, and 5 (4.9%) thought they were infected in daily life or community environment.

Of the 87 staff who thought they were infected by the working environment in hospital found, 64 (73.6%) had close contact with confirmed patients, 15 (17.2%) had close contact with suspected patients, and 36 (41.4%) were exposed to their confirmed colleagues (Fig. 1c).

Among 103 infected staff, 46 (44.7%) had worked more than seven hours a day in an environment that posed a risk of them becoming infected (Fig. 1d). The top three perceived infection routes were: droplet transmission, contact transmission, and aerosol transmission (Fig. 1e).

#### Infection-causing procedures

For nurses, the top three perceived infection causing procedures were sputum suction care, basic nursing, and pharyngeal swab collection (swab of throat) (Fig. 1f). For doctors, the top three procedures were physical examination, tracheal intubation, and manual ventilation before intubation (Fig. 1g).

#### Infection associated with protective equipment

Forty-three staff perceived protective equipment problems as the cause of their infection. Of them, 44.2% believed infection was caused by inadequate provision of protective equipment, and another 32.6% considered it was due to insufficient protection provided by the personal protective equipment (PPE) they had (wearing only surgical mask to contact confirmed cases).

#### Use of protective equipment before COVID-19 outbreak

Table 2 shows healthcare staff’s retrospective account of the level of use protective equipment in their routine work before this outbreak. 77.7% staffs always strictly followed hand hygiene, and 53.4% always strictly followed the procedure for wearing and removing of protective equipment. Among 103 staff, 66.0% always wore masks and 51.5% wore gloves in their routine work. The utilization of more sophisticated protective equipment in routine work was no higher than that of common protective equipment. We found there were 51.5, 43.7, and 50.5% of them who never used protective face shield/screen, protective clothing, and shoe covers in routine work, respectively.

**Table 2:** Comparison of the use of protective equipment between HRDs and LRDs before confirmation [*n* (%)]

Protective behaviors	Group	Never	Occasionally	Sometimes	Often	Always	P^*^
Wear mask	Total	1(1.0)	9(8.7)	15(14.6)	10(9.7)	68(66.0)	< 0.001
	LRDs	1(1.4)	9(12.7)	15(21.1)	9(12.7)	37(52.1)	
	HRDs	0(0)	0(0)	0(0)	1(3.1)	31(96.9)	
Wear glove	Total	2(1.9)	14(13.6)	19(18.5)	15(14.6)	53(51.5)	< 0.001
	LRDs	2(2.8)	14(19.7)	16(22.5)	11(15.5)	28(39.4)	
	HRDs	0(0)	0(0)	3(9.4)	4(12.5)	25(78.1)	
Strictly follow hand hygiene	Total	0(0)	2(1.9)	2(1.9)	19(18.5)	80(77.7)	0.64
	LRDs	0(0)	1(1.4)	1(1.4)	15(21.1)	54(76.1)	
	HRDs	0(0)	1(3.1)	1(3.1)	4(12.5)	26(81.3)	
Strictly follow procedures of wearing and removing	Total	11(10.7)	8(7.8)	15(14.6)	14(13.6)	55(53.4)	< 0.001
	LRDs	11(15.5)	8(11.3)	13(18.3)	11(15.5)	28(39.4)	
	HRDs	0(0)	0(0)	2(6.3)	3(9.4)	27(84.4)	
Wear protective face shield/ screen	Total	53(51.5)	7(6.8)	10(9.7)	7(6.8)	26(25.2)	< 0.001
	LRDs	48(67.6)	5(7.0)	6(8.5)	3(4.2)	9(12.7)	
	HRDs	5(15.6)	2(6.3)	4(12.5)	4(12.5)	17(53.1)	
Wear protective clothing	Total	45(43.7)	14(13.6)	10(9.7)	5(4.9)	29(28.2)	< 0.001
	LRDs	42(59.2)	10(14.1)	4(5.6)	3(4.2)	12(16.9)	
	HRDs	3(9.4)	4(12.5)	6(18.8)	2(6.3)	17(53.1)	
Wear protective shoe covers	Total	52(50.5)	7(6.8)	10(9.7)	10(9.7)	24(23.3)	< 0.001
	LRDs	45(63.4)	5(7.0)	5(7.0)	5(7.0)	11(15.5)	
	HRDs	7(21.9)	2(6.3)	5(15.6)	5(15.6)	13(40.6)	

*LRDs* Low risk of nosocomial infection department, *HRDs* High risk of nosocomial infection departments. *P value from Wilcoxon rank-sum test for two independent samples

Table 2 also demonstrated the use of protective equipment in LRDs and HRDs. In routine work, utilization of masks (96.9% vs. 52.1%) and gloves (78.1% vs. 39.4%), strict hand hygiene procedure (81.3% vs. 76.1%), and wearing and removing protective equipment (84.4% vs. 39.4%) in HRDs were much higher than those in LRDs. For more sophisticated protective equipment, the utilization in routine work in HRDs was also much higher than those in LRDs.

**Fig. 2:**
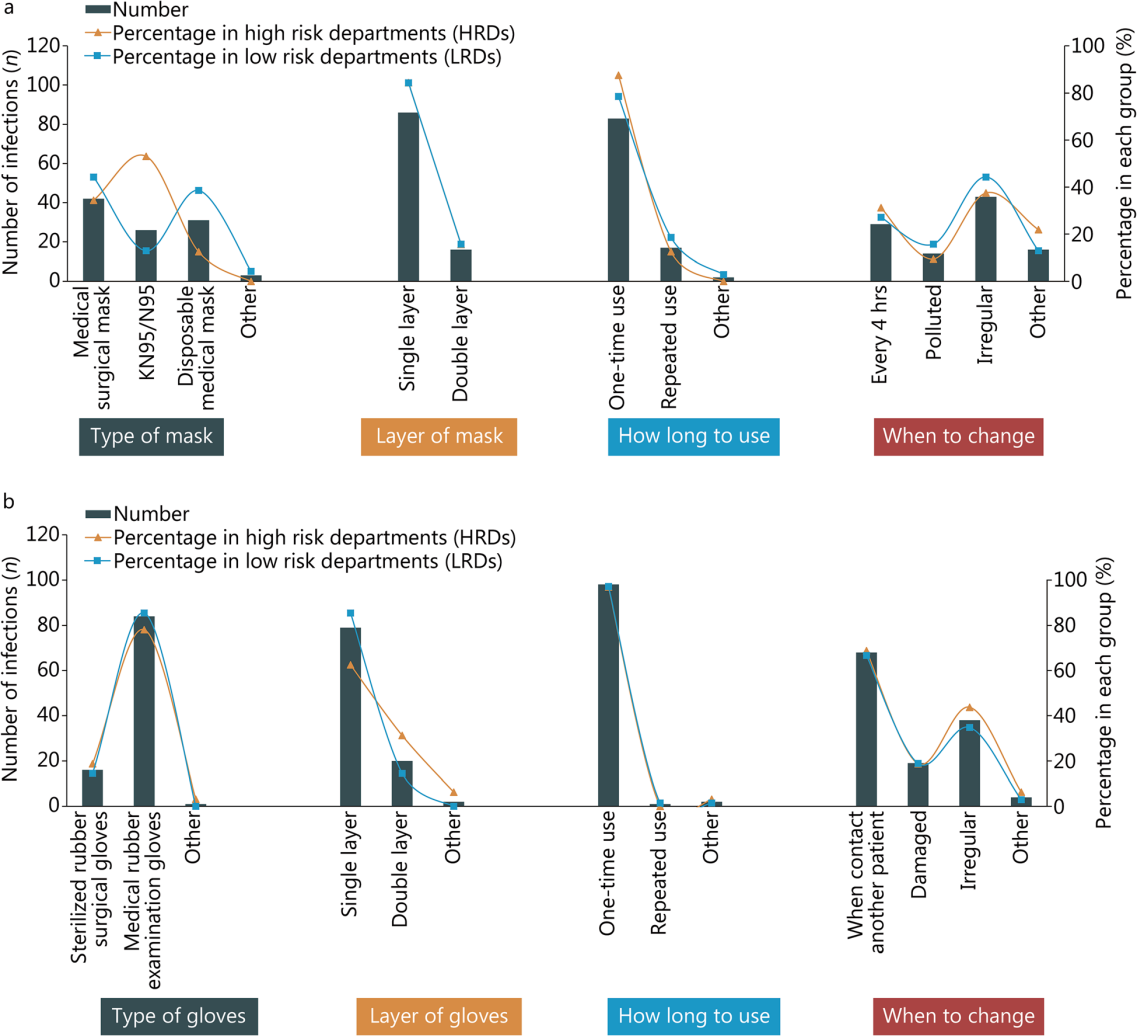
Use of protective equipment before COVID-19 outbreak. **a**. Types, layers, and length of masks usage by all participants (frequency), LRDs and HRDs (percentage) in their routine work. **b**. Types, layers, and length of gloves usage by all participants (frequency), LRDs and HRDs (percentage) in their routine work

Figure 2a and b presented types, layers, and length of masks and gloves usage by all participants (frequency), LRDs and HRDs (percentage). In their routine work, medical surgical masks were more often used than other types, and staffs in LRDs were more likely to use medical and disposable surgical masks. However, those in HRDs tended to use KN95 and N95 respirators. Medical rubber examination gloves were used frequently. Single layer and one-time use of both mask and glove were the main choices.

### Clinical symptoms, diagnosis and treatment of infected staff

#### Clinical symptoms before diagnosis

**Fig. 3:**
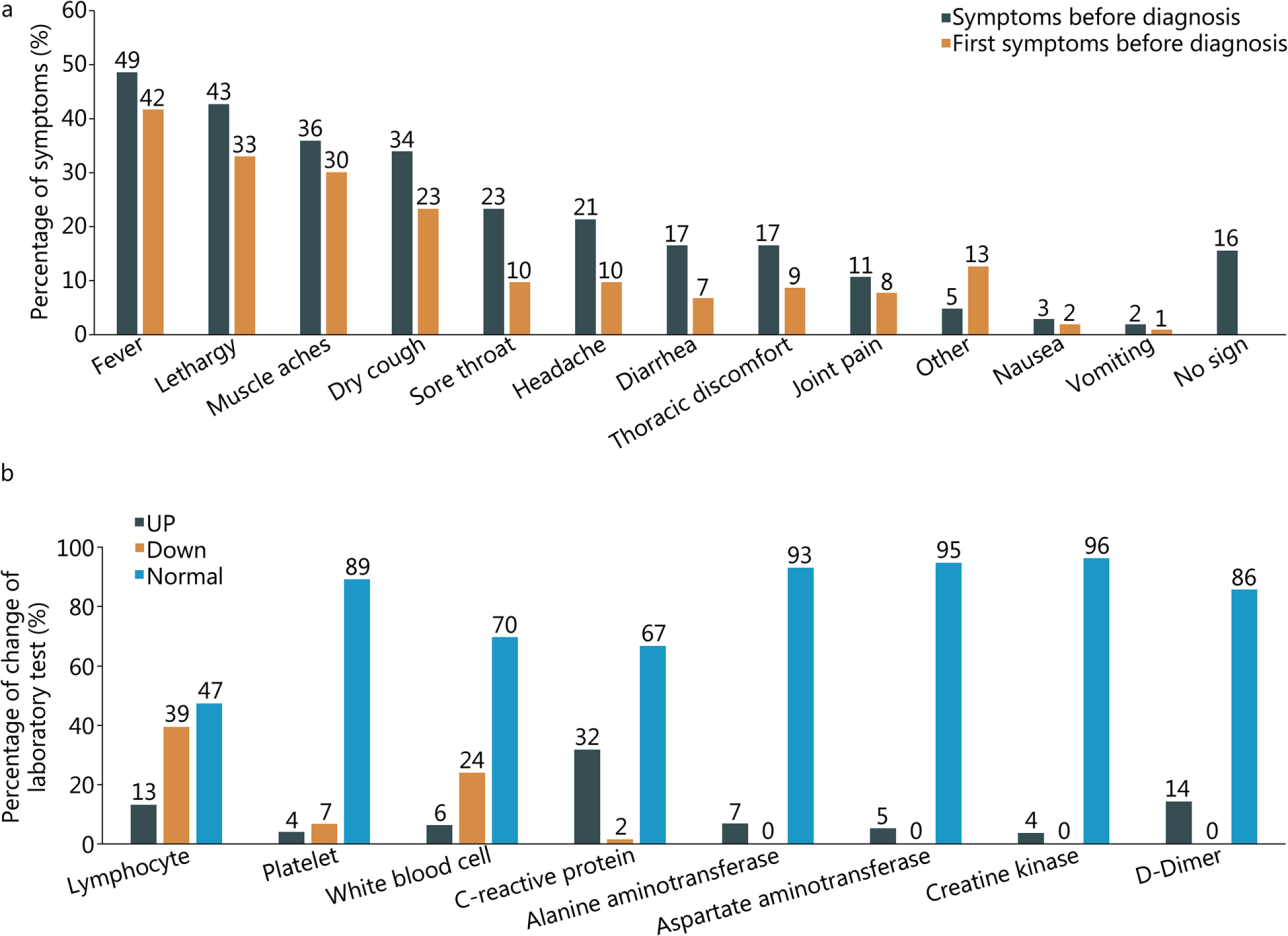
Clinical symptoms and change of laboratory indexes **a**. Clinical symptoms of all included staff before diagnosis. **b**. Laboratory and CT examination of all included staff

Of the 103 responders, symptoms were fever (48.5%), lethargy (42.7%), muscle aches (35.9%), and dry cough (34.0%) with a few of them having gastrointestinal symptoms (such as diarrhea, nausea and vomiting). In addition, the top three initial symptoms displayed before diagnosis were fever (41.8%), lethargy (33.0%), and muscle aches (30.1%), see Fig. 3a.

#### Laboratory and CT examination

As shown in Fig. 3b, among the infected cases who knew their results, 39.5% had decreased lymphocytes and 24.1% reported decreased white blood cell, but 31.8% reported increased C-reactive protein. Platelet, alanine aminotransferase, aspartate aminotransferase, creatine kinase, and D-dimer remained almost normal.

A total of 102 (99.1%) staff had CT chest examination, of which 82 were abnormal. All the 103 (100%) infected ones had nucleic acid test and 82.0% had at least one positive result or at least one suspected positive result; however, 18.0% of them had negative results.

#### Treatment after diagnosis

When confirmed as COVID-19, all the 103 cases were divided into 2 classes, of which 98 cases presented with mild/moderate clinical stages and 5 were severe clinical stages, we had on one presenting as a critical stage.

In mild/moderate cases, 12 of them received oxygen therapy, 86 received antiviral therapy, 23 received traditional Chinese medicine (TCM) treatment, three developed into severe or critical stages after admission, but none were transferred to ICU before the study closed. Among the 86 staff receiving antiviral treatment, the most common antiviral drug was oseltamivir; some also received interferon, lopinavir / ritonavir and ribavirin, but none received peramivir. The majority of them received Arbidol (Umifenovir; an anti-influza drug used in China; 69.2%), followed by immunoglobulin (19.2%) and vitamin C (16.4%).

All eight severe/critical stage cases received hormone and antibiotic treatment, three received interferon, three received antiviral therapy, one received noninvasive mechanical ventilation, and none of them received TCM treatment, extracorporeal membrane oxygenation (ECMO), or endotracheal intubation.

### Protection after infection

All of 103 cases were isolated immediately after diagnosis: 47 were in the designated hospital, 38 at home, six at centralized isolation points, and 12 in other places (hotel, infection department in hospital and a mixture).

Of the 38 who isolated at home, 76.3% wore a mask at home, 89.5% did not go out during the isolation period, and 71.1% changed their masks in less than 8 h, 68.4% of them lived alone during home isolation, most took their temperature regularly at home, washed and disinfected their hands frequently, and had independent eating utensils.

Of the 65 who were not isolated at home, 95.4% received medical treatment immediately after diagnosis, and 98.5% were not accompanied by their family members during hospital isolation; 96.9% of them thought their quarantine location was disinfected daily, and 98.5% were satisfied with the isolation environment.

### Prevention and control knowledge training before and after the outbreak

#### Before the outbreak

Of the 103 responders, 78 knew the COVID-19 transmission route; and knew most of prevention and control information of COVID-19 (with a correct rate > 90%), whereas the accuracy rate for “The window should be closed tightly in the general ward” (usually more than one patients in one sickroom in China) was only 57.3% (Table 3).

**Table 3:** Perceived correct rate of knowledge about controlling nosocomial infections before the outbreak

Number	Item	Correct rate [*n* (%)]
Item 1	Patients diagnosed with COVID-19 infection pneumonia should be concentrated and isolated	94(91.3)
Item 2	Hand hygiene should be done before putting on a mask	98(95.2)
Item 3	People should wear protective clothing in designated areas	98(95.1)
Item 4	Sputum aspiration and tracheotomy are the high-risk procedures for COVID-19 infection	99(96.1)
Item 5	The window should be closed tightly in the general ward	59(57.2)
Item 6	When contacting the patient’s blood, body fluids, secretions, excrement, vomitus and pollutants: people should wear clean gloves, and wash hands after removing the gloves	96(93.2)
Item 7	When in danger of being splashed by blood, body fluid, secretion, etc.: people should wear medical protective mask, goggles, and impermeable isolation clothing	96(93.2)

#### Comparison before and after outbreak

Compared with before outbreak, there was a focus on “Wearing protective clothing”, “Wearing goggles or face shield”, “Isolation of suspected infectious patients”, and “Wearing isolation clothes”. The training in these areas was significantly intensified (*P* < 0.05). Conversely the training intensity of “Hand hygiene” (98.1% before vs. 92.2% after) and “Wearing gloves” (96.1% before vs. 90.3% after) was significantly decreased somewhat after outbreak. (Table 4).

**Table 4:** Training rate for controlling nosocomial infections before and after the outbreak

Training for controlling nosocomial infections	Training rate [*n* (%)]		P^*^
	Before outbreak	After outbreak	
Isolation of suspected infectious patients	76(73.8)	90(87.4)	0.004
Environmental cleaning and disinfection	86(83.5)	91(88.4)	0.17
Hand hygiene	101(98.1)	95(92.2)	0.03
Wearing gloves	99(96.1)	93(90.3)	0.03
Wearing surgical mask	90(87.4)	92(89.3)	0.56
Wearing goggles or face shield	71(68.9)	89(86.4)	< 0.001
Wearing isolation clothes	81(78.6)	90(87.4)	0.02
Wearing protective clothing	66(64.1)	90(87.4)	< 0.001

^*^P value from McNemar test

Compared with before outbreak, the level of awareness of information related to “Isolation of suspected infectious patients”, “Environmental cleaning and disinfection”, “Wearing goggles or face shield”, “Wearing protective clothing”, and “Wearing isolation clothes” has been significantly increased since the outbreak (*P* < 0.001), while the high level of awareness of “Hand hygiene” and “Wearing gloves” were only slightly enhanced (Table 5).

**Table 5:** Comparison of mastery level of training for controlling nosocomial infections before and after the outbreak [*n* (%)]

Training for controlling nosocomial infections	Very unfamiliar	Not familiar	Neutral	Familiar	Very familiar	P^*^
Isolation of suspected infectious patients						< 0.001
Before outbreak	12(11.7)	4(3.9)	16(15.5)	25(24.3)	46(44.7)	
After outbreak	6(5.8)	2(1.9)	7(6.8)	20(19.4)	68(66.0)	
Environmental cleaning and disinfection						< 0.001
Before outbreak	5(4.9)	6(5.8)	16(15.5)	21(20.4)	55(53.4)	
After outbreak	5(4.9)	2(1.9)	8(7.8)	16(15.5)	72(69.9)	
Hand hygiene						0.65
Before outbreak	1(1.0)	0(0)	7(6.8)	11(10.7)	84(81.6)	
After outbreak	4(3.9)	0(0)	2(1.9)	12(11.7)	85(82.5)	
Wearing gloves						0.69
Before outbreak	1(1.0)	1(1.0)	7(6.8)	11(10.7)	83(80.6)	
After outbreak	5(4.9)	0(0)	3(2.9)	11(10.7)	84(81.6)	
Wearing surgical mask						0.17
Before outbreak	3(2.9)	1(1.0)	9(8.7)	16(15.5)	74(71.8)	
After outbreak	4(3.9)	1(1.0)	3(2.9)	10(9.7)	85(82.5)	
Wearing goggles or face shield						< 0.001
Before outbreak	9(8.7)	8(7.8)	13(12.6)	16(15.5)	57(55.3)	
After outbreak	7(6.8)	2(1.9)	3(2.9)	11(10.7)	80(77.7)	
Wearing isolation clothes						< 0.001
Before outbreak	6(5.8)	6(5.8)	14(13.6)	19(18.5)	58(56.3)	
After outbreak	6(5.8)	3(2.9)	4(3.9)	14(13.6)	76(73.8)	
Wearing protective clothing						< 0.001
Before outbreak	15(14.6)	5(4.9)	14(13.6)	22(21.4)	47(45.6)	
After outbreak	8(7.8)	1(1.0)	7(6.8)	18(17.5)	69(67.0)	

^*^P value from Wilcoxon signed-rank test for paired samples

#### Improvements for prevention and control of nosocomial infections

Results of these staffs’ opinions regarding improvements needed for fighting nosocomial infection showed that: 84.5% chose “Medical staff protection”, followed by “Emergency plan and work flow” (68.0%) and “Pay attention to the health of Medical staff” (66.0%); More than half believed further attention was needed in “Infection outbreak management”, “Full staff training”, “Patient visit management”, “Division of infection risk areas”, and “Infection monitoring”.

### Psychological status

**Fig. 4:**
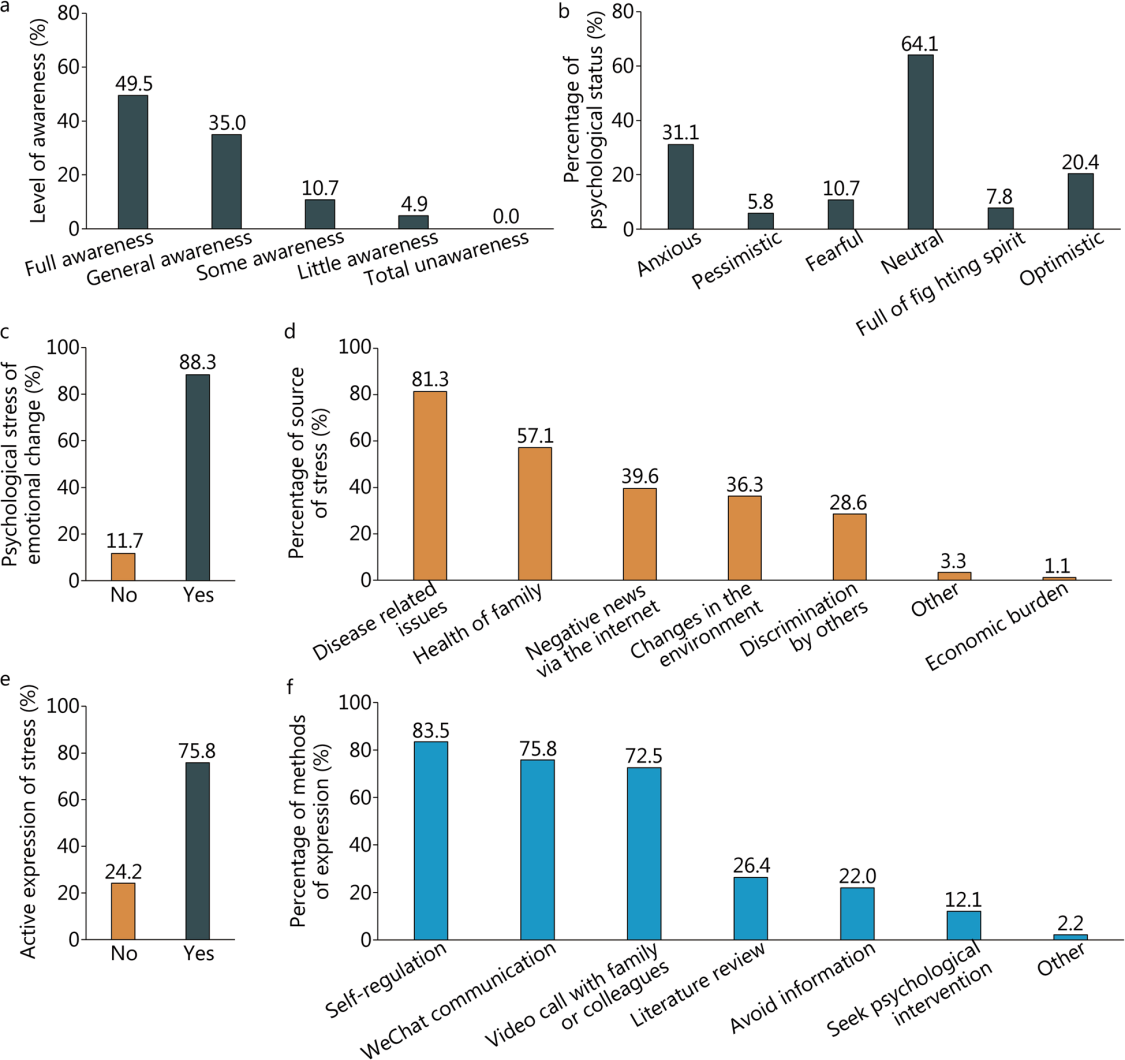
Psychological statuses before and after diagnosis of COVID-19 **a**. Attention level to the epidemic before their diagnosis; **b**. Mental status since the outbreak; **c**. Psychological stress or emotional changes during their isolation; **d**. Reasons of psychological stress and emotional change; **e**. Willingness to express psychological stress or emotional changes; **f**. Methods to regulate their stress or mood changes; **a-b**, psychological status before diagnosis; **c-f**, psychological status after diagnosis

Figure 4 presents their psychological status. Before they were diagnosed with infection, 49.5% said that they were fully aware of the seriousness of the situation, 64.1% remained neutral, 31.1% were anxious, 20.4% maintained an optimistic attitude, and only a few were fearful or pessimistic.

After diagnosis, 88.3% staff experienced psychological stress or emotional changes during their isolation period, only 11.7% had almost no emotional changes. Of the 88.3% ones, their psychological stress or emotional changes were caused by the disease related issues (81.3%), worried about their families’ health (57.1%), negative internet news (39.6%); and only 1.1% were worried about the economic burden.

Fortunately, they all took effective measures to control their emotions or stress, 75.8% staffs had actively expressed their psychological stress. The ways they used include self-regulation, communicating with others on WeChat, and video call with family or colleagues.

## Discussion

Protecting medical professionals from infection is crucial to success in fighting COVID-19. However, cluster outbreaks occurred in many departments in this study, for 9 departments had more than five cases. More than half of the infected staff had close contact with confirmed or suspected patients in their department or work environment before they knew the true status of SARS-CoV-2.

During early January 2020, patients having fever and coughs were scatted throughout many hospitals across Wuhan, just when influenza and common pneumonia rates are high and they were all treated in the same way. At that time most medical staffs usually used ordinary medical masks without any other medical equipment during these patients’ diagnosis and treatment [15]. Reports showed human-to-human transmission of SARS-CoV-2 was confirmed by the infection of 15 healthcare professionals after close contact with infected patients in a Wuhan hospital [16]. For nearly a month, neither the public nor healthcare professionals were clear about all the characteristics of COVID-19. During that time many medical staffs were not taking enhanced personal protective measures. Our results were confirmed repeatedly by cross-checking information from the departments’ directors of infected hospital workers and participants. Lack of effective protective measures during the early stage is an important risk factor, and the main reason for transmission was failure to protect people working at a close distance and having intimate contact with infected patients. The prevention and control plan before diagnosis can help to explain this. Only a fifth of infected staffs were routinely equipped with protective face shields, clothing or shoe covers. Most of them just wore the usual protective equipment. In addition, improper use of PPE (e.g., repeated use of masks) is also a factor involved in increasing the infection risk [4].

With the continuous occurrence of confirmed infections during January 2020, the hospitals and government of Wuhan introduced strategies and policies to improve the management of patients and protection of medical staff. The personal protection awareness of medical staff increased rapidly and the number of confirmed cases decreased rapidly during February 2020. We conducted an in-depth telephone interviews for cases confirmed in February 2020. For infected healthcare professionals beyond the concentrated outbreak period in January 2020, we cannot rule out that they may have experienced a longer incubation period before they were diagnosed as COVID-19. In addition, some confirmed cases had worked in the frontline for a long time maybe leading to a lowered resistance to infection. The interview information showed that the last case we included is a frontline nurse who had been engaged in combating COVID-19; she claimed that she was fully equipped by uniform standards at work, and she had frequently conducted CPR for confirmed patients before her own infection was confirmed. She believed that she was infected by confirmed patients, but was not sure about exactly when or which patient was involved. Moreover, the first group of infected medical staff also forms part of the chain of transmission of nosocomial infections. 39 cases in our study suggested that their route of infection may be “close contact with colleagues who were subsequently”, but they were infected during the early stage of epidemic.

Generally, we believe that physical examination poses a relatively lower infection risk than tracheal intubation or tracheotomy, but physical examination was selected by the highest proportion of infected doctors. In our study, nine infected doctors in the hepatopancreatobiliary surgery department, which is a LRD for nosocomial infection, reported that physical examination is the most likely cause of their infection. From tracking their questionnaire data we found that inadequate protective equipment and insufficient protection of protective equipment were frequently selected. The percentage of staff selecting “personal protective equipment used more often” was higher in HRDs than LRDs in routine work. This is maybe the reason why more infected cases appeared in LRDs. However, we can only hypothesize why this occurred, because normally people don’t use sophisticated infection control procedures routinely in LRDs and to do so would be poor use of resources and also frightening for the public.

As the above emphasizes, the main mode of transmission was failure to maintain protection when working at a close distance and having intimate contact with infected cases. For epidemics, like COVID-19, protection for low exposure risk procedures should be upgraded, and medical protective masks, protective face shield/screens and protective clothing are also needed especially in the emergency or infectious diseases department or when caring for high risk patients [12].

Training in knowledge and skills for prevention and control COVID-19 for healthcare providers is important [10], especially as professor Chen Wang believes that SARS-CoV-2 may turn into a chronic disease and coexist with humans like the flu [17].

Therefore, in order to cope with the long-term existence of SARS-Cov-2 and possible public health emergencies in future, health care workers should receive annual training on the use of personal PPE and additional education during surge events [4].

The learning and dissemination of epidemic prevention knowledge and skills should be increased not only for medical areas but for all the public. The areas of personal protection, emergency planning and work flow should be improved.

Shortages of PPE that intensify fears of coronavirus exposure at work contribute to the psychological distress or other illness. Stable and adequate PPE supply is highly recommended even the time without pandemic. Administrators in hospital mustensure that their staff feel safe, respected, prepared, and supported [4]. In addition, basic rules should be stressed, such as emphasizing hand hygiene, installing barriers to limit contact with patients at triage, prioritizing respirators for aerosol generating procedures and self-monitoring for signs of illness and self-isolating and reporting illness to managers, if it occurs [18, 19].

To sustain and restore frontline health care workers, health care organizations need to monitor the mental health outcomes of healthcare personals over time and prioritize the mental and physical health needs and recovery of first-line healthcare professionals [20]. Understanding how workers are exposed to the virus will be essential to protect frontline healthcare staff. But, little knowledge is known about the presence of the virus contamination on surfaces in hospitals and concentration of virus in the air. This lack of evidence means we are using a precautionary approach which often results in our applying all available controls all the time. It also means that resources are used unnecessarily, possibly leading to shortages for workers in particular environments where they could provide protection [21]. Research about better ways of protecting workers from the virus is still needed.

The major limitation of this study is estimating the exact reason of infection for some cases is not an easy thing, and some memory biases maybe exist among participants. Participants usually pondered over the causes of infection. They could overstate the protection measures they were using before their diagnosis because they are reluctant or unwilling to believe that this is how they got infected.

## Conclusions

Effective protective measures were generally lacking in hospitals in the early stage of COVID-19. The main factor affecting transmission was not using protecting equipment when working at a close distance and having intimate contact with infected persons. Most staff experienced psychological stress or emotional changes during their isolation period a after diagnosed. Protective equipment should be upgraded in hospital at the onset of a new disease especially for staff conducting procedures involving close contact and caring for high risk patients. Also, learning from these lessons is expected to help the Chinese government and other parts of the world to better respond to future unexpected infectious disease outbreaks.

## Supplementary information

**Additional file 1.** Appendix 1 Questionnaire.

## Data Availability

The datasets used and/or analyzed during the current study are available from the corresponding author on request.

## Abbreviations

CNHC: The National Health Committee of the People’s Republic of China
COVID-19: Coronavirus disease 2019
ECMO: Extracorporeal membrane oxygenation
HRDs: High risk of nosocomial infection departments
LRDs: Low risk of nosocomial infection departments
PPE: Personal protective equipment
SARS-CoV-2: Severe acute respiratory syndrome coronavirus 2
TCM: Traditional Chinese medicine
