# Supplementary material for: Perceived infection transmission routes, infection control practices, psychosocial changes, and management of COVID-19 infected healthcare workers in a tertiary acute care hospital in Wuhan: a cross-sectional survey
Source: Mil Med Res. 2020 May 11;7:24. doi: 10.1186/s40779-020-00254-8 (PMC7211983; doi:10.1186/s40779-020-00254-8)
Supplement: Supplementary file 1 — Additional file 1. Appendix 1 Questionnaire. [file 40779_2020_254_MOESM1_ESM.docx]

**Perceived infection transmission routes, infection control practices, psychosocial changes, and management of COVID-19 infected healthcare professionals in a tertiary acute care hospital in Wuhan**

**Informed Consent**

**Affiliation and Contact Information:**

Dear colleagues,

At present, COVID-19 infection presents a great challenge for all health care staff. A study on the optimization of protection strategies for the COVID-19 infection among health care staff has been launched, and I tis supported by the research group on prevention and treatment of pneumonia caused by COVID-19 in Zhongnan Hospital of Wuhan University. Your support and cooperation will be greatly appreciated.

The results of this study will be used for scientific research only, i.e. the prevention and control strategies for major epidemics in hospitals. This survey covers the routes of infection, measures of prevention and control, treatment and outcomes of pneumonia. Your personal information will be desensitized. If you agree to participate in the survey, please click on “Agree” and help us fill out this questionnaire,

We are looking forward to your support, thank you!

**I voluntarily agree to participate in this research program**

**□ Yes**

**□ No**

**Signature ________________________________ Date ______________**

**Basic information**

**1. Name: ________**

**2. Gender:**

**A.** Male

**B.** Female

**Are you pregnant?**

A. Yes

B. No

**3. Age: ________ years**

**4. Height: ________ cm**

**5. Weight: ________KG**

**6. Marital status:**

A. Married

B. Remarried

C. Cohabiting

D. Single

E. Widowed

F. Divorced

**7. Nationality/ethnic group:**

A. Han Chinese

B. non-Han, ________ *(please enter the nationality/ ethnic group)*

**8. Work unit:** ________ (*please enter your work unit, not the designated hospital where your epidemic prevention work is located)*

**9. What is your condition after the infection?**

A. I have recovered and been discharged from hospital.

B. I have returned to work in an epidemic prevention unit

C. I am being treated in hospital.

D. I am being isolated at home.

E. other, ________

**9.1. If you were working in an epidemic prevention unit, please select the hospital where you are now:**

A. Mobile cabin hospital in Wuhan

B. Leishenshan Hospital in Wuhan

C. Wuhan No.7 Hospital

D. Zhongnan Hospital of Wuhan University

E. Other, ________

**10. What is your job?**

A. Doctor

B. Nurse

C. Medical technician, ________

**11. How long have you been working there:** ________Years

**12. In what capacity/level are you working?**

A. Senior level

B. Associate Senior level

C. Intermediate level

D. Junior level

**13. Which department are you working in?** ________

**14. Your risk factors and comorbidity**

| Chronic heart disease, *including congenital heart disease (excluding hypertension)* | 🞎Yes 🞎No 🞎NA | Obesity *(BMI>28)* | 🞎Yes 🞎No 🞎NA |
| --- | --- | --- | --- |
| Hypertension | 🞎Yes 🞎No 🞎NA | Diabetes with complications | 🞎Yes 🞎No 🞎NA |
| Chronic lung disease *(excluding asthma)* | 🞎Yes 🞎No 🞎NA | Diabetes without complications | 🞎Yes 🞎No 🞎NA |
| Asthma | 🞎Yes 🞎No 🞎NA | Rheumatic diseases | 🞎Yes 🞎No 🞎NA |
| Chronic kidney disease | 🞎Yes 🞎No 🞎NA | Malnutrition | 🞎Yes 🞎No 🞎NA |
| Moderate or severe liver disease | 🞎Yes 🞎No 🞎NA | Smoking | 🞎Yes 🞎No 🞎Quit Smoking |
| Mild liver disease | 🞎Yes 🞎No 🞎NA | Alcohol consumption | 🞎Yes 🞎No 🞎Quit drinking |
| Chronic nervous system disease | 🞎Yes 🞎No 🞎NA | Other risk factors: __________ | |
| Malignant tumor | 🞎Yes 🞎No 🞎NA |  |  |
| Chronic hematological diseases | 🞎Yes 🞎No 🞎NA |  |  |
| AIDS / HIV | 🞎Yes 🞎No 🞎NA |  |  |

***NA,*** *Not Applicable*

**15. Do you Smoke?**

A. Yes

B. No

C. Quit Smoking

**16. Do you drink alcohol?**

A. Yes

B. No

C. Quit drinking

**17. Have you been cured?**

A. No

B. Yes, time from my diagnosis to cure):________ days

**18. Do you have any previous experience in treatment and nursing of a major epidemic, e.g. SARS?**

A. Yes

B. No

**Main Text**

**Part 1. Before diagnosis**

**(I) Analysis of Causes of Infection**

**1. Which is the most likely route of your infection?**

A. Daily life / community environment

B. Working environment in hospital

C. Laboratory environment (biological laboratory, clinical lab, etc.) with biological specimens of suspected or confirmed cases

D. Other, ________

E. I don’t yet know how I got infected

**1.1 If you choose “A. daily life / community environment”, I think the route of my infection may be: (multiple choices)**

A. Have been to a place with cases infected by COVID-19, e.g. Huanan Seafood Market.

If you choose this, please provide the place name: ________

B. Have close contact with confirmed or suspected cases of COVID-19.

C. Have had direct contact with wild animals.

If you choose, please provide name of the wild animals: ________

D. Have attended large parties or visited crowded places, e.g. train stations, shopping malls, etc.

E. Other, ________

*"Close contact" is defined as: a. close contact or studying and working in the same room with patients with the novel coronavirus infection; b. Have taken any form of transportation with patients with the novel coronavirus infection; c. Lived with patients with the novel coronavirus infection.*

**1.2 If you choose “B. Working environment in hospital”, please answer the following two questions.**

**1.2.1 I think the route of my infection may be: (multiple choices)**

A. Had close contact with confirmed patients in my department or work environment.

B. Had close contact with suspected patients in my department or work environment.

**Sub-Question: do you know whether the suspected patient has been confirmed?**

A. Yes

B. No

C. Unclear

D. Had close contact with a feverish patient with a high risk of COVID-19 (Our team will to verify whether the patient is diagnosed as suspected, confirmed, or not COVID-19).

**1.2.2Do you know whether the feverish patient has been confirmed?**

A. Yes

B. No

C. Unclear

D. Had close contact with confirmed colleagues in department or work environment.

E. Other, ________

| *Notation: "Close contact" is defined as: a. Health care-related occupational exposure, including direct contact with patients infected with the novel coronavirus, such as health care workers and their colleagues who are treating the infected patients, visiting the patients, close contact with the patients, or direct exposure to the patients’ body fluid, samples, or droplets; b. Close contact or studying and working in the same room as patients with the novel coronavirus infection; C. Have taken any form of transportation with patients with the novel coronavirus infection; D. Lived with patients with the novel coronavirus infection.* |
| --- |

**1.2.3What kind of "close contact" do you think fits your route of infection? (multiple choices)**

A. Direct contact with patients infected with the novel coronavirus, such as visiting the patients, having close contact with the patients, or direct exposure to the patients’ body fluid, samples, or droplets.

B. Have worked with health care staff who are treating the patient.

C. Have taken any form of transportation with the infected patients.

D. Other, ________

**1.3 If you choose “C. Laboratory environment”, please answer the questions.**

**1.3.1: I think the route of my infection may be: (multiple choices)**

A. Contact with COVID 19 confirmed biological specimens in the laboratory

（If you choose this option, please continue to answer with which of the following specimens did you have contact? a. Secretions b. Blood c. Urine d. Feces e. Alveolar lavage fluid f. sputum g. Not sure）

B. Contact with suspected biological specimens in the laboratory

（If you choose this option, please continue to answer with which of the following specimens did you have contact? a. Secretions b. Blood c. Urine d. Feces e. Alveolar lavage fluid f. sputum g. Unsure h. Not sure）

C. Although I have not come into contact with confirmed or suspected biological specimens in the laboratory, I have been in the same room where there were confirmed or suspected biological specimens.

(If you choose this option, please continue to answer with which of the following specimens did you have contact? a. Secretions b. Blood c. Urine d. Feces e. Alveolar lavage fluid f. sputum g. Not sure）

D. Other,

**2. Do you think the most likely route of your infection is: (Multiple choices)**

A. Droplet transmission

B. Contact transmission

C. Aerosol transmission (e.g. tracheal intubation, non-invasive ventilation, tracheotomy, cardiopulmonary resuscitation, etc.)

D. Digestive tract transmission ¨

E. Other,

F. Not sure

**3. If you are a nurse, please choose the procedures that you think is most likely to cause the infection:** **(Multiple choices)**

A. None

B. Sputum suction care

C. Atomized inhalation

D. Tracheotomy care

E. Arteriovenous puncture

F. Tracheal intubationcare

G. Pharyngeal swab specimen collection

H. Collection of specimens other than pharyngeal swabs

I. Basic nursing procedures such as turning over a patient

J. Cardiopulmonary resuscitation (CPR)

K. ECMO-related nursing operations

L. Other,

**4. If you are a doctor, please choose the proceedure that you think is most likely to cause the infection: *(multiple choices) (the doctor answers)***

A. None

B. Tracheal intubation

C. Noninvasive ventilation

D. Making the tracheotomy

E. Cardiopulmonary resuscitation

F. Manual ventilation before intubation

G. ECMO-related operations

H. Bronchoscopy

I. Physical Examination

J. Other,

**5. How many hours a day were you at risk of becoming infected before your diagnosis?** **(Exposure to the environment that you think might be the source of your infection):**

A. less than 1h

B. about 1-2h

C. about 3-6h

D. about 7-10h

E. more than 10h

**6. In addition to the above, what other pathways do you think can lead to the infection of COVID-19 in medical staff? *(Open, optional)***

|  |
| --- |

**II. Prevention and control plan before COVID-19 outbreak**

**1. Did you routinely wear a mask during medical or nursing work before** **COVID-19 outbreak?**

A. Always

B. Often

C. Sometimes

D. Occasionally

E. Never

**If the answer is not “Never", please continue to answer the following 4 questions**

**1.1Type of masks:**

A. Medical surgical mask

B. KN95 / N95 and above particulate matter protective mask or medical protective mask

C. Disposable medical mask

D. Other,

**1.2 Layers of masks worn:**

A. Single layer (with one mask)

B. Double layer (with two at the same time)

C. Other,

**1.3 Frequency of use of masks:**

A. One-time use

B. Repeated use

C. Other,

**1.4 Replacement frequency of masks:**

A. Every 4 hours

B. After pollution

C. Irregular

D. Other

**2.** **Did you wear gloves when you were engaged in medical or nursing work beforeCOVID-19 outbreak?**

A. Always

B. Often

C. Sometimes

D. Occasionally

E. Never

**If the answer is not "Never", please continue to answer the following 4 questions**

**2.1 Type of gloves:**

A. Sterilized rubber surgical gloves

B. Medical rubber examination gloves

C. Other,

**2.2 Layers of gloves worn:**

A. Single layer (with 1 pair of gloves)

B. Double layer (wear two pairs of gloves at the same time)

C. Other,

**2.3** **How long did you use a pair of gloves?**

A. One time

B. Repeatedly

C. Other,

**2.4 How often did you change gloves?**

A. When contacting different patients

B. When they became damaged

C. Irregularly

D. Other,

**3. Did you wear goggles or a protective face shield / screen for work involving close contact with patients’ body fluids when there was exposure risk beforeCOVID-19 outbreak?**

A. Always

B. Often

C. Sometimes

D. Occasionally

E. Never

**If the answer is not "never", please continue to answer the following questions**

**3.1 Frequency of disinfection:**

A. When contacting different patients

B. When they are polluted

C. Sometimes

D. Other, _________

**4.** **Did you wear protective clothing for medical or nursing work when there wasexposure risk beforeCOVID-19 outbreak?**

A. Always

B. Often

C. Sometimes

D. Occasionally

E. Never

**If the answer is not “Never", please continue to answer the following questions:**

**4.1 Types** **of protective clothing:**

A. Conforms to national standard（GB19082-2009）

B. Conforms to Japanese, American, European and other standards

C. Other, _________

**4.2** **layers worn:**

A. Monolayer (i.e., single-piece)

B. Bilayer (i.e., two-piece or wearing isolation gown in addition to protective clothing)

C. Other, _________

**4.3 Frequency of use：**

A. Disposable

B. Reusable

C. Other, _________

**4.4 Frequency of Replacement**

A. When in contact with different patients

B. When they are polluted

C. When they are damaged

D. Sometimes

E. Other, _________

**5. Did you wear protective shoe covers, rubber boots or, waterproof boot covers for work involving patient contact when you were exposed to risk before COVID-19 outbreak?**

A. Always

B. Often

C. Sometimes

D. Occasionally

E. Never

**6. Did you strictly implement hand hygiene measures in medical or nursing work before COVID-19 outbreak?** *(Please note that this means using the seven steps hand washing procedure when you wash your hands).*

A. Always

B. Often

C. Sometimes

D. Occasionally

E. Never

**7. D****id you know and strictly follow the procedures for wearing and taking off protective equipment for health care staff during medical or nursing work beforeCOVID-19 outbreak?**

A. Always

B. Often

C. Sometimes

D. Occasionally

E. Never

**8. If you think the cause of your infection may be related to protective equipment, please answer the following questions:**

**8.1 What do you think is the most important cause of infection related to protective equipment?**

A. Improper use of protective equipment (For example, did not wear an isolation gown according to the procedure, re-use single-use mask, mask did not cover nose etc.)

B. Insufficient protection (For example, wore a medical surgical mask to contact the confirmed patients closely, but the mask was not replaced after more than 4 hours, etc.)

C. Broken protective clothing or defective quality (For example, clothing failed to meet national standards.)

D. Not enough protective equipment

E. Failure to be protected for a short time：In the process of diagnosing and treating someone, a confirmed or suspected patient forcibly increased your risk of infection, (for example, they remove your mask or spit on you, etc.）

F. Other, _________

**8.2** **Which kind of protective equipment did you used improperly in actual practice?**

A. None

B. Masks

C. gloves

D. Isolation gown

E. Goggles

F. Other, _________

**8.3 Why do you think the above protective equipmentwas used improperly?**

A. The wearing procedure is not standard

B. The removing procedure is not standard

C. Overuse of protective equipment

D. Other, _________

**8.4 What kind of protective equipment do you think gives insufficient protection?**

A. None

B. Masks

C. Gloves

D. Isolation gown

E. Goggles

F. Other, _________

**8.5 What do you think is the reason for the insufficient protection provided by the above protective equipment?**

A. Protective equipment does not meet the national standard

B. Wearing insufficient layers

C. Not replaced often enough

D. Other, _________

**III. Infection Prevention and control knowledge before** **COVID-19 outbreak**

**1. Did you know the transmission route of COVID-19 infected pneumonia before your diagnosis?**

A. Yes

B. No

**2. Please judge whether the following** **statements are correct?**

| Patients diagnosed with COVID-19 infection pneumonia should be concentrated and isolated | 🞎Right🞎Wrong □Don't know |
| --- | --- |
| Hand hygiene should be done before putting on a mask | 🞎Right🞎Wrong □Don't know |
| People should wear protective clothing in designated areas | 🞎Right🞎Wrong □Don't know |
| Sputum aspiration and tracheotomy are the high-risk operations forCOVID-19 infection | 🞎Right🞎Wrong □Don't know |
| The window should be closed tightly in the general ward | 🞎Right🞎Wrong □Don't know |
| When contacting the patient's blood, body fluids, secretions, excrement, vomitus and pollutants: people should wear clean gloves, and wash hands after removing the gloves | 🞎Right🞎Wrong □Don't know |
| When in danger of being splashed by blood, body fluid, secretion, etc.: people should wear medical protective mask, goggles, and impermeable isolation clothing | 🞎Right🞎Wrong □Don't know |

**3. Have you received training in infection control in hospitals and the mastered operating skills beforeCOVID-19 outbreak?**

|  | yes or no | Mastery level (5 is very unfamiliar, 1 is very familiar) |
| --- | --- | --- |
| Isolation of suspected infectious patients | 🞎 yes 🞎 no | 5🞎 4🞎 3🞎 2🞎 1🞎 |
| Environmental cleaning and disinfection | 🞎 yes 🞎 no | 5🞎 4🞎 3🞎 2🞎 1🞎 |
| Hand hygiene | 🞎 yes 🞎 no | 5🞎 4🞎 3🞎 2🞎 1🞎 |
| Wearing gloves | 🞎 yes 🞎 no | 5🞎 4🞎 3🞎 2🞎 1🞎 |
| Wearing surgical mask | 🞎 yes 🞎 no | 5🞎 4🞎 3🞎 2🞎 1🞎 |
| Wearing goggles or face shield | 🞎 yes 🞎 no | 5🞎 4🞎 3🞎 2🞎 1🞎 |
| Wearing isolation clothes | 🞎 yes 🞎 no | 5🞎 4🞎 3🞎 2🞎 1🞎 |
| Wearing protective clothing | 🞎 yes 🞎 no | 5🞎 4🞎 3🞎 2🞎 1🞎 |
| Other, | 🞎 yes 🞎 no | 5🞎 4🞎 3🞎 2🞎 1🞎 |

**4.** **How did you get the training in preventing hospital infection? (Multiple choice)**

A. Training at your hospital

B. Training at other hospitals

C. National special training

D. Self-study online

E. Other

**5. Have you received any training related to hospital infection control and mastered operating skills sinceCOVID-19 outbreak?**

|  | yes or no | Mastery level (5 is very unfamiliar, 1 is very familiar) |
| --- | --- | --- |
| Isolation of suspected infectious patients | 🞎 yes 🞎 no | 5🞎 4🞎 3🞎 2🞎 1🞎 |
| Environmental cleaning and disinfection | 🞎 yes 🞎 no | 5🞎 4🞎 3🞎 2🞎 1🞎 |
| Hand hygiene | 🞎 yes 🞎 no | 5🞎 4🞎 3🞎 2🞎 1🞎 |
| Wearing gloves | 🞎 yes 🞎 no | 5🞎 4🞎 3🞎 2🞎 1🞎 |
| Wearing surgical mask | 🞎 yes 🞎 no | 5🞎 4🞎 3🞎 2🞎 1🞎 |
| Wearing goggles or face shield | 🞎 yes 🞎 no | 5🞎 4🞎 3🞎 2🞎 1🞎 |
| Wearing isolation clothes | 🞎 yes 🞎 no | 5🞎 4🞎 3🞎 2🞎 1🞎 |
| Wearing protective clothing | 🞎 yes 🞎 no | 5🞎 4🞎 3🞎 2🞎 1🞎 |
| Other, | 🞎 yes 🞎 no | 5🞎 4🞎 3🞎 2🞎 1🞎 |

**6.** **What do you think is correct when performing procedures that may produce aerosols for suspected or confirmed patients (such as tracheal intubation, noninvasive ventilation, tracheotomy, cardiopulmonary resuscitation, manual ventilation prior to intubation and bronchoscopy, etc.)? (Multiple choice)**

A. Take air isolation measures

B. Wearing medical protective masks and testing for tightness

C. Eye protection (such as goggles or masks)

D. Wear long-sleeve gowns that prevent body fluid penetration and gloves

E. perform medical procedures in a well-ventilated room

F. The number of people in the room should be limited and only those who care and support the patient need to be present

**IV.Psychological response to the epidemic before diagnosis**

**1. Had you been paying enough attention to this epidemic before your diagnosis?**

A. Fully aware of gravity of situation

B. Generally aware

C. Some understanding of the situation

D. Little awareness of the situation

E. Totally unaware of the situation

**2. What has been your mental attitude since COVID-19 outbreak? (Multiple choices)**

A. Neutral

B. Anxious

C. Pessimistic

D. Fearful

E. Full of fighting spirit

F. Optimistic

**Part 2. After diagnosis**

**I. Symptoms after diagnosis**

**1. Did you have the following symptoms before your diagnosis? (Multiple choices)**

A. None

B. Fever; **If fever, maximum body temperature: (________) ℃**

C. Dry cough

D. Diarrhea

E. Vomiting

F. Lethargy

G. Sore throat

H. Nausea

I. Headache

J. Thoracic discomfort

K. Muscle aches

L. Joint pain

M. Other,

**2. The first symptoms before your diagnosis were? (Multiple choice)**

A. Fever, **If fever, maximum body temperature: (________) ℃**

B. Dry cough

C. Diarrhea

D. Vomiting

E. Lethargy

F. Sore throat

G. Nausea

H. Headache

I. Thoracic discomfort

J. Muscle aches

K. Joint pain

L. Other,

**3. Were there any abnormalities in your chest X-ray examination at the time of your diagnosis?**

A. Yes

B. No

C. CXR not done

**4. Were there any abnormalities in your chest CT scan at the time of your diagnosis?**

A. Yes

B. No

C. CT scan not done

**5. Were you tested for nucleic acid at the time of your diagnosis?**

A. Yes（If yes, please answer: negative or positive）

B. No

**6. Which of the following indicators presented abnormal results during laboratory inspection?**

| Lymphocyte | 🞎↑ 🞎↓  🞎Normal🞎Unclear | Alanine aminotransferase(ALT) | 🞎↑ 🞎↓  🞎Normal🞎Unclear |
| --- | --- | --- | --- |
| Platelets | 🞎↑ 🞎↓  🞎Normal🞎Unclear | Aspartate aminotransferase(AST) | 🞎↑ 🞎↓  🞎Normal🞎Unclear |
| White blood cell(WBC) | 🞎↑ 🞎↓  🞎Normal🞎Unclear | Creatine kinase(CK) | 🞎↑ 🞎↓  🞎Normal🞎Unclear |
| C-reactive protein(CRP) | 🞎↑ 🞎↓  🞎Normal🞎Unclear | D-Dimer | 🞎↑ 🞎↓  🞎Normal🞎Unclear |
| 🞎 Other, | | | |

**II. Treatment after diagnosis**

**1. How severe was your infection at diagnosis?**

A. Mild

B. Moderate

C. Severe

D. Critical

| *Notation:1）Mild：clinical symptoms are mild, and no pneumonia manifestations on imaging examination；2）Moderate：fever and respiratory related symptoms, pneumonia on imaging examination；3)Severe：Meet one of the following conditions:①respiratory distress and RR ≥ 30 times / min；②finger resting oxygen saturation ≤ 93%；③PaO_2_/FiO_2_ ≤ 300mmHg；4）Critical：Meet one of the following conditions:①respiratory failure occurs and mechanical ventilation is required；②shock；③combined with other organ failure and requires ICU monitoring and treatment.* |
| --- |

**If you choose Mild or Moderate, please continuing to answer the following questions.**

1.1 Did this progress to Severe or Critical after hospitalization?

A. Yes

B. No

**2. Did you receive oxygen therapy？**

A. Yes

B. No

**If you choose “Yes”, which of the following administrative methods were used? (Multiple choice)**

A. Nasal catheter

B. Oxygen mask

C. High flow nasal oxygen therapy (HFNO)

D. Non-invasive ventilation (NIV)

E Invasive mechanical ventilation

F. Other, __________

**3. Did you receive antiviral treatment?**

A. Yes

B. No

**If you choose “Yes”, which of the following drugs were used? (Multiple choice)**

A. Interferon

B. Lopinavir/ritonavir

C. Ribavirin

D. Oseltamivir

E. Zanamivir

F. Peramivir

G. Other, __________

**4. Did you have a bacterial infection?**

A. Yes

B. No

**If you choose “Yes”, did you have antibiotic treatment?**

A. Yes, __________

B. No

**5. Did you receive Traditional Chinese medicine treatment?**

A. Yes

B. No

**If you choose “Yes”, what kind of Traditional Chinese medicine treatment did you have? __________**

**6. Did you receive the following drugs during the treatment? (Multiple choice)**

A. Convalescent plasma

B. Vitamin C

C. Immunoglobulin

D. Remdesivir

E. Arbidol

F. Other________

G. I don’t know

**7. Did you have the following complications during or after treatment?**

A. Acute respiratory distress syndrome (ARDS)

B. Septic Shock

C. Acute renal injury

D. Disseminated intravascular coagulation (DIC)

E. Other, __________

**8. Were you transferred to ICU during the treatment?**

🞎 Yes, **the length of stay in ICU is (________) days.**

🞎 No

**9. If your condition was severe or critical, or progressed from mild or moderate to severe or critical, what kind of drugs or treatments did you receive in the severe stage? (Multiple choice)**

A. Hormone

B. Interferon

C. Antiviral drugs

D. Antibiotics

E. Neuromuscular blocker

F. Vasopressin or positive inotropic drugs

G. Traditional Chinese medicine treatment

H. non-invasive ventilation (NIV)

I. invasive mechanical ventilation

J. Extracorporeal membrane oxygenation (ECMO)

K. Hemodialysis / Hemofiltration

L. Prone position ventilation

M. Tracheostomy and catheterization

N. Other, __________

**Ⅲ. Impact on environment and people after infection.**

**1. Were you isolated immediately after diagnosis? (Isolation at home or hospital within 24 hours after diagnosis)**

A. Yes

B. No

**2. Was your diagnosis reported by your head of department or infection management professional to center of disease control based on related rules in China?**

A. Yes

B. No

C. I don’t know

| *Note: When finding suspected cases, clinical diagnosis cases, confirmed cases and asymptomatic infected persons, all kinds of medical institutions at all levels should report directly online within 2 hours. After receiving the report, the CDC should immediately investigate and verify, and complete the three-level confirmation audit of the report information through the network direct reporting system within 2 hours.* |
| --- |

**3. Is your family currently infected? (from your diagnosis up to now)? Do they have:-**

A. Any suspected patients*,* **how many? (________ )**

B. Any confirmed patients, **how many? (________ )**

C. All healthy

D. I don’t know

**4. If you were isolated after diagnosis, where were you isolated?**

A. Isolated at home

B. Isolated at a designated hospital

C. Isolated at a centralized isolation point

D. Isolated at other places______ *place name is required if other places were selected*

**4.1 If you choose isolated at home, please answer these questions**

**4.1.1 Did you wear a mask at home?**

A. Yes

B. No

**4.1.2 Did you go out during the isolation period?**

A. Yes

B. No

**4.1.3 If you went out, did you wear a mask?**

A. Yes

B. No

**4.1.4 How often did you change the mask?**

A. ≤ 4h

B. 5-8h

C. 9-12h

D. ≥13h

**4.1.5 When you were isolated at home, were you living alone or with your family?**

A. Alone

B. Lived with your family

**4.1.6 Did you take your temperature regularly at home?**

A. Yes

B. No

**4.1.7 Did you wash and disinfect your hands frequently at home (more times than before diagnosis)?**

A. Yes

B. No

**4.1.8 If you lived with your family during the isolation period, did you have independent eating utensils at home?**

A. Yes

B. No

**4.2 If you were not isolated at home, please answer these questions**

**4.2.1Who were you in contact with after diagnosis** *(contact refers to close conversation whether wearing masks or not, shaking hands, dining together, face-to-face conversation)*

A. Family members

B. Colleagues

C. Friends

D. Medical staff

E. Other, ________

**4.2.2 Did you get medical treatment immediately after diagnosis?**

A. Yes

B. No

C. Uncertain

**4.2.3 In which area of the designated hospital were you isolated?**

A. Fever outpatient

B. Emergency

C. Respiratory ward

D. Intensive care ward

**4.2.4 Did you have family members accompanying you during the hospital isolation?**

A. Yes

B. No

**4.2.5 Is your isolated location disinfected daily?**

A. Yes

B. No

**4.2.6 Are you satisfied with your isolated environment then or now?**

A. Yes

B. No，what are you dissatisfied with? _________

**IV. Awareness of post-infection protection**

**1. Based on your professional perspective and experience of this infection and treatment, what part of the hospital protection needs to be improved during the outbreak of infectious diseases? (Multiple choice)**

A. Emergency plan and work flow

B. Full staff training

C. Medical staff protection

D. Pay attention to the health of medical staff

E. Infection monitoring

F. Cleaning and disinfection management

G. Patient visit management

H. Patient education

I. Infection outbreak management

J. Medical waste management

K. Other,

**2. If you have some suggestions for improvement of hospital protection, please write them down.**

|  |
| --- |

**V. Psychology after diagnosis**

**1. Did you experience any psychological stress or emotional changes during the isolation period? ("0" means almost no emotional change, and "5" means great psychological pressure or emotional change)**

A. 0

B. 1

C. 2

D. 3

E. 4

F. 5

**If you did not choose “0”, please answer this question:**

**1.1 What are the possible causes of your emotional change? (Multiple choices)**

A. Disease related issues

B. Economic burden

C. Discrimination by others

D. Changes in the environment

E. Health of family

F. Negative news via the internet

G. Other,

**1.2 Are you willing to discuss your psychological stress or emotional changes?**

A. Yes

B. No

**1.3 As a health care worker, which do you usually choose to regulate your stress or mood changes?**

A. Communicate with others on WeChat

B. Video call with family or colleagues

C. Seek professional psychological crisis intervention (online, video, telephonehelp lines, etc.)

D. Read recent literature on this disease

E. Self-regulation

F. Avoid considering information about this outbreak

G. Other,

**2. What are the sources of comfort and care available to you after the infection? (Multiple choice)**

A. Partner (lover)

B. Parents

C. Children

D. Brothers and sisters

E. Leaders and colleagues

F. News reports

G. Counseling professional or general

Thank you very much for your great participation!

**Would you like to recommend your colleagues to participate in the survey?**

A. Yes

B. No

*(Thank you very much for your support. In order to further explore the infection causes of medical staff and provide strong evidence for epidemic prevention and control, the research team needs you to recommend non-infected people who work with you for a further comparative study. We sincerely hope that you can select qualified colleagues according to the following criteria to participate in the follow-up research)*

**Please recommend three of your colleagues to participate in this survey according to the following criteria:**

*1) Non-infected;*

*2) In the same administrative office or the same department of epidemic prevention;*

*3) The responsibilities of job are basically the same as yours (if you were probably infected by an infected patient, please recommend colleagues who were also involved in the diagnosis and treatment of this patient).*

**Please give us the name and phone number of your colleagues:**

1.

2.

3.
